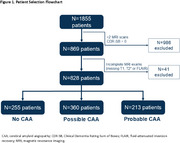# Identifying and Characterizing Comorbid Cerebral Amyloid Angiopathy in ADNI Participants using Boston Criteria Version 2.0

**DOI:** 10.1002/alz70856_098357

**Published:** 2025-12-24

**Authors:** Robert W Deering, Neal S Parikh, Sasikiran Goteti, Ying He, Luc Bracoud, Andreja Avbersek, Joseph Chiarappa, Farshid Sepehrband

**Affiliations:** ^1^ Alnylam Pharmaceuticals, Inc., Cambridge, MA, USA; ^2^ Clario, Inc., Lyon, France; ^3^ Regeneron Pharmaceuticals, Inc., Tarrytown, NY, USA

## Abstract

**Background:**

Cerebral amyloid angiopathy (CAA) is characterized by progressive cerebrovascular amyloid‐beta deposition associated with hemorrhagic and nonhemorrhagic manifestations. Evidence of prior cerebral hemorrhage and CAA can have significant implications for treatment with some approved therapies. This study sought to estimate the proportion of Alzheimer's Disease Neuroimaging Initiative (ADNI) participants who have probable CAA by systematically applying Boston Criteria version 2.0. In addition, we sought to evaluate characteristics associated with probable CAA in this population.

**Method:**

Participants in ADNI with cognitive symptoms or deficits (at least one Clinical Dementia Rating‐Sum of Boxes [CDR‐SB] score of >0) with relevant imaging for CAA adjudication were included. 3D T1‐weighted, fluid‐attenuated inversion recovery (FLAIR) and T2* Gradient Echo magnetic resonance imaging (MRI) sequences were downloaded from the ADNI database. Blinded neuroradiologists evaluated brain MRIs to categorize participants as having no CAA, possible CAA, or probable CAA using the Boston Criteria version 2.0. A 10% sample was randomly selected to evaluate inter‐reader variability. Baseline characteristics associated with probable CAA were assessed using univariate and multiple regression analyses.

**Result:**

828 participants met inclusion criteria (Figure 1): 30.8% had no CAA, 43.5% had possible CAA, and 25.7% had probable CAA. Among those with probable CAA, 46.9% had mild cognitive impairment and 44.6% had dementia; median CDR‐SB score was 3 (IQR 1‐5). Among age, sex, CDR‐SB score, *apolipoprotein E4* (*APOE4*) homozygosity, hypertension, and antithrombotic medication use, an exploratory multiple logistic regression identified older age, higher CDR‐SB score, and *APOE4* homozygosity as independently and significantly associated with probable CAA (*p* <0.05). High interobserver agreement was observed (inter‐reader correlation >90%). Additional results will be presented.

**Conclusion:**

Systematic application of Boston Criteria version 2.0 using the ADNI database identified probable CAA in approximately 26% of participants with cognitive symptoms or deficits. Age, dementia severity, and *APOE4* genotype may inform the likelihood of having CAA; however, probable CAA was identified in patients across the spectrum of cognitive impairment severity. These data underscore the value of rigorous assessment of CAA in patients with cognitive complaints broadly, and the need for research and clinical paradigms to meet the needs of such patients.